# New treatments for progeria

**DOI:** 10.18632/aging.102626

**Published:** 2019-12-22

**Authors:** Ricardo Villa-Bellosta

**Affiliations:** 1 Fundación Instituto de Investigación Sanitaria, Fundación Jiménez Díaz (FIIS-FJD), Universidad Autónoma de Madrid, 28040 Madrid, Spain

**Keywords:** aging, treatment, ATP, pyrophosphate, vascular calcification

Hutchinson-Gilford Progeria Syndrome (HGPS) is an extremely rare and fatal autosomal dominant genetic condition that causes accelerated aging in children. Typically, those born with progeria live to their mid-teens or early twenties. HGPS is caused by accumulation of progerin (a protein altered during normal aging) at the nuclear envelope. Most people with HGPS harbor a single nucleotide substitution (GGC→GGT; G608G) within exon 11 of the gene encoding lamin A [1,2]. This mutation activates a cryptic splice-donor site, leading ultimately to a truncated, permanently farnesylated isoform of prelamin A (progerin) that results in a dimorphic nuclear phenotype in HGPS. Reversion of the HGPS mutation in mice using CRISP-Cas9 extends longevity, highlighting a potential therapeutic approach [1,2]. However, this treatment strategy is a long way from being applicable in humans. Therefore, improving the main symptoms, which stem from production and accumulation of progerin, is the main treatment strategy for this devastating disease [3,4].

**Figure 1:**
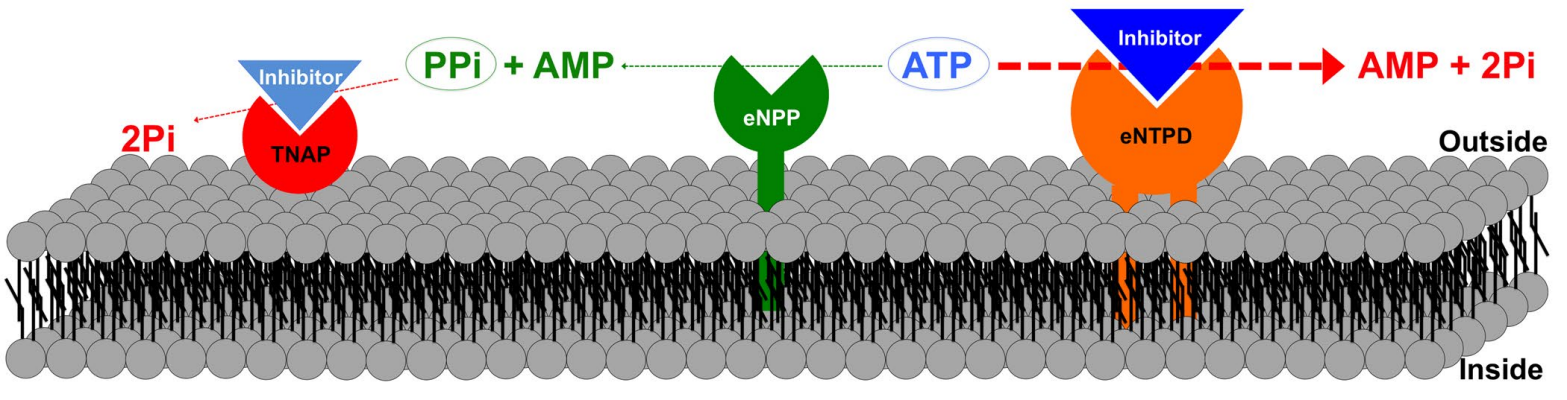
**Schematic representation of extracellular pyrophosphate metabolism**. Ectonucleotide pyrophosphatase phosphodiesterase (eNPP) hydrolyzes adenosine triphosphate (ATP) to release adenosine monophosphate (AMP) and pyrophosphate (PPi). By contrast, ectonucleoside triphosphate diphosphohydrolase (eNTPD), hydrolyzes ATP to release AMP and phosphate (Pi). Pyrophosphate is degraded to phosphate by tissue non-specific alkaline phosphatase (TNAP). Inhibition of eNTPD and TNAP activities may increase availability of both ATP and pyrophosphate.

However, a recent study describes an alternative strategy for treatment of HGPS [5]. As in humans, mice with HGPS display excessive vascular calcification, a common clinical manifestation associated with aging, diabetes, and chronic kidney disease. This incremental increase in calcium deposition on the aortic wall in HGPS mice is due to deficiency of extracellular pyrophosphate [6], a potent endogenous inhibitor of calcification [7]. This reduction in extracellular pyrophosphate levels in HGPS mice is a consequence of impaired synthesis of pyrophosphate, both in the aorta and blood, which in turn is caused by three factors. The first is up-regulation of tissue non-specific alkaline phosphatase (TNAP), the main enzyme involved in pyrophosphate degradation (Figure 1). The second is up-regulation of ectonucleoside triphosphate diphosphohydrolase (eNTPD), an enzyme that hydrolyzes ATP to release two phosphates plus AMP (Figure 1). The third is reduced production of ATP (the source of pyrophosphate) due to mitochondrial dysfunction associated with reduced complex IV activity [5,6].

Pyrophosphate is produced by hydrolysis of extracellular ATP via eNPP (ectonucleotide pyrophosphatase/phosphodiesterase; Figure 1); loss of eNPP function results in generalized arterial calcification during infancy, which is characterized by calcification of the arteries [8]. Moreover, a study in HGPS mice shows that extracellular ATP plays a critical role both as a source of pyrophosphate and as a direct inhibitor of vascular calcification [5]. Notably, eNTPD activity (ATP→phosphate) in the aorta and blood is dominant over eNPP activity (ATP→pyrophosphate). More than 90% of extracellular ATP is hydrolyzed to release phosphate; therefore, less than 10% of the hydrolyzed extracellular ATP generates pyrophosphate in the aorta and blood. This is enhanced by loss of plasmatic eNPP activity [5].

Reduced ATP production in addition to pyrophosphate degradation and reduced pyrophosphate synthesis (due to an increase in the eNTPD/eNPP ratio) in HGPS mice leads to a marked reduction in availability of pyrophosphate [5,6]. *Ex vivo*, combined inhibition of eNTPD and TNAP increases pyrophosphate availability both in the aorta and blood, and prevents calcification of the aortic wall [5]. ATP replacement therapy prevents vascular calcification without affecting the life span of HGPS mice. By contrast, combined treatment with ATP and inhibitors of TNAP and eNTPD increases longevity and prevents vascular calcification [5]. Although vascular calcification is dependent on plasma levels of ATP and pyrophosphate, combined treatment with ATP and eNTPD/TNAP inhibitors may increase availability of ATP in local tissue, thereby providing more energy for maintenance of life.

This new treatment strategy for HGPS could constitute an alternative therapy for this devastating syndrome. In addition, it may provide an alternative to eNPP replacement therapy to restore extracellular pyrophosphate levels in diseases caused by pyrophosphate deficiency [8].
